# Didactic qualification of teaching staff in primary care medicine – a position paper of the Primary Care Committee of the Society for Medical Education

**DOI:** 10.3205/zma001346

**Published:** 2020-09-15

**Authors:** Klaus Böhme, Irmgard Streitlein-Böhme, Erika Baum, Horst Christian Vollmar, Markus Gulich, Maren Ehrhardt, Folkert Fehr, Bert Huenges, Barbara Woestmann, Ralf Jendyk

**Affiliations:** 1 Ruhr-Universität Bochum, Abteilung für Allgemeinmedizin und Familienmedizin, Bochum, Germany; 2 Universität Marburg, Abteilung für Allgemeinmedizin, präventive und rehabilitative Medizin, Marburg, Germany; 3 Universitätsklinikum Ulm, Institut für Allgemeinmedizin, Ulm, Germany; 4 Universitätsklinikum Hamburg-Eppendorf, Institut und Poliklinik für Allgemeinmedizin, Hamburg, Germany; 5 Facharzt für Kinderheilkunde und Jugendmedizin, Sinsheim an der Elsenz, Germany; 6 Westfälische Wilhelms-Universität Münster, Centrum für Allgemeinmedizin, Münster, Germany

**Keywords:** primary care medicine, medical didactics, qualification teaching staff

## Abstract

Having teaching staff with didactic qualifications in university teaching leads to a measurable improvement in academic skills among students. Previous recommendations on the type and scope of medical didactic qualification measures primarily apply to teaching staff at university and in-patient settings. The situation of primary care medicine, which often employs external lecturers and whose teaching takes place to a considerable extent in decentralized training facilities (teaching practices) is not adequately addressed.

Taking into account a survey on the status quo at higher education institutions for General Practice in Germany, recommendations for minimum standards are made, based on national and international recommendations on the content and scope of medical didactic qualification measures.

These recommendations include preliminary work by the Personnel and Organizational Development in Teaching (POiL) Committee of the Society for Medical Education (GMA), the MedicalTeachingNetwork (MDN), the Society of University Teaching Staff in General Medicine (GHA) as well as the experiences of the committee members, who hail from the field of general medicine, internal medicine and pediatrics amongst others.

## Zusammenfassung

Die didaktische Qualifizierung von Lehrenden im Bereich der Hochschullehre führt zu einer messbaren Verbesserung akademischer Kompetenzen bei Studierenden. Bisherige Empfehlungen zu Art und Umfang von medizindidaktischen Qualifizierungsmaßnahmen erstrecken sich im Wesentlichen auf Lehrende im universitären und stationären Setting. Dabei wird auf die Situation der primärversorgenden Medizin, die häufig auf externe Lehrbeauftragte zurückgreift und deren Lehre in erheblichem Umfang in dezentralen Ausbildungsstätten (Lehrpraxen) stattfindet nicht hinreichend eingegangen.

Ausgehend von nationalen und internationalen Empfehlungen zu Inhalt und Umfang medizindidaktischer Qualifizierungsmaßnahmen werden hier – unter Berücksichtigung einer Umfrage zum Status Quo an den deutschen allgemeinmedizinischen Hochschulabteilungen – Empfehlungen für Mindeststandards ausgesprochen.

Eingegangen in diese Empfehlungen sind Vorarbeiten des Ausschusses „Personal- und Organisationsentwicklung in der Lehre“ (POiL) der „Gesellschaft für Medizinische Ausbildung“ (GMA), des „MedizinDidaktikNetzes“ (MDN) , der „Gesellschaft der Hochschullehrer für Allgemeinmedizin“ (GHA) sowie die Erfahrungen der Ausschussmitglieder, die u.a. den Gebieten Allgemeinmedizin, Innere Medizin und Kinderheilkunde angehören.

## Introduction

The underlying messages of empirical research into teaching and learning is that workplace training in didactics in higher education comprehensively increases the teaching skills of lecturers, which in turn leads to a measurable improvement in the acquisition of academic skills among students [1]. Since the development of mature teaching staff is a very complex and heterogeneous process, measures for the sustainable improvement of teaching competences have very different starting points. For the medical didactics sector, two BEME guides (No. 8 & No. 40) were able to show that a multitude of different programs for medical teaching staff internationally have lasting positive effects [2], [3].

The Personnel and Organizational Development in Teaching (POiL) Committee was founded as part of the Society for Medical Education (GMA) in 2003, initially with the aim of developing and establishing didactic qualification measures at the university level [4]. In Germany there is also the MedicalTeachingNetwork (MDN), which is a working group of the Association of Medical Faculties (MFT), an association of all medical faculties that design and implement didactic qualification offers for the professionalization of higher education teaching staff in medicine. In 2012, it agreed on formal and substantive minimum standards for didactic qualification offers throughout Germany [5].

However, all national and international activities aiming to improve teaching in medicine have in common that they essentially refer to the qualifications of teaching staff in university and in-patient settings. This does not address medical training in General Practice in particular, as this frequently relies on external lecturers and decentralized training facilities such as general medical practices (teaching practices). Other subject areas in primary care using similar models in training will be similarly affected. 

In a previous position paper by the Primary Care Committee [6], which essentially dealt with questions of the structural and process quality of teaching in primary care medicine, questions of didactic qualification were only touched upon, so these are to be examined in more detail here.

## Description of the situation in Germany

In an inventory of higher education didactic qualification measures throughout the German-speaking countries, the GMA POiL Committee 2006 found that the offering ranged from isolated events in the form of individual courses on various didactic topics at the faculty level to and university-wide programs to entire master’s degree programs with extensive qualification options [7]. 

When formulating requirement profiles, the Committee for Medical Teaching Staff recommends comprehensive modular qualification programs with a minimum of 200-240 teaching units (UE). Face-to-face events (courses, workshops), to a certain extent short events (lectures, seminars), course presentations as practical projects, internships and practical advice, project learning in the sense of teaching project outlines, self-study, teaching portfolios as well as mentoring and on-the-job learning are seen as suitable formats [8]. 

The recommended course contents and thematic priorities can be found in table 1 (Tab. 1) below.

In addition, personality development, self- and role-reflection as well as aspects of self- and time-management should be discussed [8].

In addition, the Primary Care Committee believes that the aspect of faculty development plays an important role in the qualifications of full-time teaching staff. This includes among other things the ability to continuously identify needs for didactic and content support, tailored to the respective target group (e.g. other full-time teaching staff, teaching physicians of the elective blocks and PY practices...), to design and implement corresponding teaching offers with a low threshold and to evaluate them. 

In a further position paper in 2015 [9], in the course of the discussion about a stronger orientation of teaching towards competences that should be acquired, the POiL Committee had already formulated the following six competence fields for medical teaching staff:

- Medical didactics
- Learner orientation
- Social and communicative competence
- Role model and professional behavior
- Reflection and further development of one’s own teaching practice
- System-related teaching and learning

When measuring the effectiveness and success of qualification offers, the POiL Committee concludes that, in addition to a high level of satisfaction with such offers, this results in teaching staff developing a more positive attitude towards teaching and a subjective and objective increase in medical didactic knowledge leading to changed behaviors in teaching [10].

In its 2015 consensus paper [5], the MDN reiterates its support for a nationwide uniform concept of medical didactic qualifications of 200 teaching units and proposes a two-stage concept in the form of a basic qualification (120 TU) and a specialization qualification (80 TU).

These quality standards propose a trainer qualification (at least one person in the trainer team with an MME degree or comparable qualification), the existence of a program/agenda, a limited group size per trainer (max. 8-10 participants), duration of the individual courses at least 24 TU, max. 10 units/day, attendance time of at least 50% of the total time, at least 50% of attendance time consisting of practical exercises, operationalized (broad) learning goals, a meaningful change of method and an active follow-up of the attendance time, which includes at least one collegial coaching event and one self-reflection event [5]. 

The substantive requirements for a qualification program correspond to those of the POiL Committee of the GMA [8].

From fall 2015 to spring 2016, the Primary Care Committee conducted a survey on the status of didactic qualifications at General Practice higher education institutions in Germany. The aim was to ascertain the status quo as the basis for later recommendations. The following data refer to this survey.

By mid-2016, 30 of 37 higher education institutions contacted had responded to the survey. The evaluation of the tables gave the following results (see table 2 (Tab. 2)).

The numerical results of the survey can be seen in table 2 (Tab. 2) and table 3 (Tab. 3). Only those locations that had reported back were included in the calculation of the range as well as the mean values. Under “Other events”, the following were mentioned among others: Meeting of lecturing doctors, General Practice Day, participation in MME courses, coaching and internships in teaching practices by higher education staff, ...

As General Practice has the most extensive experience in the use of external lecturers and teaching practices, an example of the characteristics for other subjects in primary care must be discussed with regards to the recommendations derived from the survey results. 

## Assessment and position

The recommendations made by the POiL Committee and the MDN on the didactic qualification of teaching staff in medicine in higher education are based on a careful analysis of nationally and internationally proven models. Thus, they should be just as valid for permanent employees of higher education institutions for General Practice as for employees in institutions of other primary care subjects.

The situation is different for external visiting lecturers and for teaching staff in General Practice/primary care teaching practices. In general the feasibility of implementing such standards in the primary care context should be considered. 

Based on the theory of the sociologist Everett M. Rogers on the introduction of innovation, the POiL Committee identified five points as criteria for the feasibility of introducing qualification measures that also appear to be relevant for teaching staff in primary care medicine [11], [12]:

- Complexity of the measures
- Compatibility (with everyday routines, with professional practice)
- Relative advantage (is the effort worth it?)
- Visibility (e.g. comparable to scientific achievements?)
- Testability (flexibility of the measures)

The results of the survey among higher education institutions for General Practice (siehe table 3 (Tab. 3)) about didactic qualification measures show an extremely inconsistent picture of optional and obligatory offers. The Primary Care Committee is now concerned with formulating minimum standards for the didactic qualification of teaching staff in primary care in order to create possible foundations for quality improvement or assurance. The starting point for the following recommendations is the status quo at the locations, formats already established by the Society of University Teaching Staff in General Medicine (GHA) and questions of feasibility as mentioned above.

## Recommendations of minimum standards for the didactic qualification of teaching staff in primary care medicine

Against this background, recommendations for the medical didactic qualification of teaching staff in primary care medicine – according to the varying requirement profiles and areas of application – should be differentiated for:

1. Higher education employees
2. Teaching staff in shadowing practices
3. Teaching staff in elective block practices (EB practices)
4. Teaching staff in training practices for the Practical Year (PY practices)
5. External lecturers who teach at the higher education institution

University staff should also take over the training and supervision of teaching staff in terms of 3, 4 and 5. Their qualification is therefore also to be understood in the sense of a Train-the-Trainer concept.

The contents and thematic focal points of the didactic qualifications listed below correspond to those in table 1 (Tab. 1). There are no recommendations for the content of regular didactic refresher courses, these should be determined on a case by case basis at the locations according to needs and target groups.

There should be an option for qualitatively and quantitatively higher-level qualifications such as an MME degree or a completed pedagogical degree amongst others to replace qualification measures.

### 1. Higher education employees 

For the permanent teaching staff of higher education institutions of primary care subjects, the same recommendations are made here as the MDN demands as a qualification for all teaching staff of medicine in higher education:

- **200 TU** with coverage of all content and focal points listed in table 1 (Tab. 1), and of these
- **120 TU** basic qualification
- **80 TU** specialization

Regular didactic workplace training was not addressed by the MDN. Only one higher education institution for General Practice reported these as being obligatory but without specifying the scope; 35 locations report an average of 35 TU per year on a voluntary basis. In line with the scope for visiting lecturers, the committee considers **workplace training of 2 x 4 TU** per year as appropriate.

For university employees who are deployed in the M3 exam, an **examiner workshop** of **8 TU** should be offered. This scope corresponds to a GHA format that has been in use for several years.

#### 2. Teaching staff in shadowing/internship practices

**No specific didactic qualification measures **are called for practice owners who offer shadowing/internships. The reason for this is frequent lack of links to higher education institutions and the high turnover. Alternatively, the locations (shadowing) and/or the professional societies (internships) should provide the practices with written information on training content and objectives as well as a possible structuring of shadowing/internships. 

#### 3. Teaching staff in elective block practices (EB practices)

The didactic qualification of instructors in the EB should cover the following topics based on table 1 (Tab. 1):

- Learning theory
- Lesson planning
- Small group formats (here: doctor - students - patient in clinical teaching)
- Feedback
- Examinations (optional, if EB practices carry out performance assessments)

Introductory courses on a voluntary basis were reported by 5 higher education locations for General Practice, the average size was 6 TU; at 5 locations, introductory courses with an average of **5 TU** were obligatory (see table 3 (Tab. 3)).

For many years, the GHA has offered **Introductory Courses for Teaching Staff in EB** at various locations with a scope of **14 TU**. In view of the subject areas to be covered, the committee recommends keeping this format. 

The surveyed locations on average offer 7 TU of **regular didactic workplace training events**; at 6 locations these are voluntary, at 12 obligatory. The committee recommends offering **2 x 3 TU** per year, ideally docked to the teaching practice meetings that often take place every semester.

#### 4. Teaching staff in training practices for the practical year (PY practices)

Topics to be covered:

- Learning theory
- Lesson planning
- Small group formats (here: doctor - students - patient in clinical teaching)
- Feedback
- M3 examinations

The higher education locations for General Practice reported an average of 5 TU (voluntary) to 7 TU (obligatory) for the basic qualification of PY trainers (see table 3 (Tab. 3)). The didactic basic qualification of instructors in the PY should be based on the qualification for the EB: the Committee recommends to only accredit practices for the PY that have at least two years of experience in the EB, have regularly participated in teaching practice meetings and have received positive evaluations. 

This means that **14 basic qualification TU** can be presumed. In order to adapt the training content to the needs in the PY that differ from the EB, an **advanced qualification of around 8 TU** should be offered in a modular format. This format also corresponds to a tried and tested concept that has been used by members of the Committee for some time.

For PY instructors who are deployed in the M3 exam, an **examiner workshop** of around** 8 TU** should be offered.

The locations reported an average of 5 TU (voluntary) or 4 TU (obligatory) of **regular didactic workplace training** for PY trainers (see table 3 (Tab. 3)). The committee recommends offering **4 TU** per year.

#### 5. External lecturers who teach at the higher education institution

Depending on their area of application, the didactic qualification of external lecturers should extend at least to the following subject areas:

- Learning theory
- Lesson planning
- Media
- Lecture/presentation technology
- Small group formats
- Feedback
- Examinations (optional, depending on deployment in faculty or state examinations)

The survey showed that 5 locations required obligatory introductory courses with on average 9 TU, 12 locations offered courses running to an average of 26 TU on a voluntary basis (see table 3 (Tab. 3)).

It can be assumed that most General Practice lecturers also act as instructors in EB practices. Thus, most of the subject areas would already be covered by the **basic qualification** for EB practices of **14 TU**. Knowledge of **seminars and plenary didactics** would also have to be imparted. Due to the modular structure of didactic training, a further 15 TU seem appropriate to cover these two subject areas. If deployed in the context of M3 exams, an examiner workshop of **8 TU** is also required.

Regarding **regular didactic training**, 5 locations reported 8 TU per year, 14 locations 9 TU per year on a voluntary basis (see table 3 (Tab. 3)). On this basis, the Committee recommends **2 x 4 TU per year**.

Summary of Recommendations (see table 4 (Tab. 4)).

## Summary and outlook

The present recommendations of the GMA’s Primary Care Committee for the didactic qualification of teaching staff in general and primary care medicine are based on preparatory work by the POiL Committee of the GMA and the MDN, the practical experiences of the GHA and its committee members, and a survey of the status quo in higher education institutions for General Practice in Germany.

Although one can prove the usefulness of university didactic qualification measures [1], there is no empirical evidence for the duration of such measures or the timing of refresher measures. As a rule, it was necessary to fall back on experiences with existing programs, usually running into many years. 

The recommendations made here can certainly serve as a model for subjects beyond primary care against the background of the Masterplan in Medical Studies 2020, with a significant expansion of student training in medical practices in all specialties. 

For General Practice, the didactic qualification measures for teaching staff during training which are already modular to some extent can provide a basis for the training of instructors in competence centers for further training as well as for those authorized to continue training in practices. Similarly, training in further education should also be recognized as part of specialization teaching.

## Note

The position paper was acepted by the GMY executive board at 28-05-2020.

## Competing interests

The authors declare that they have no competing interests. 

## Figures and Tables

**Table 1:**
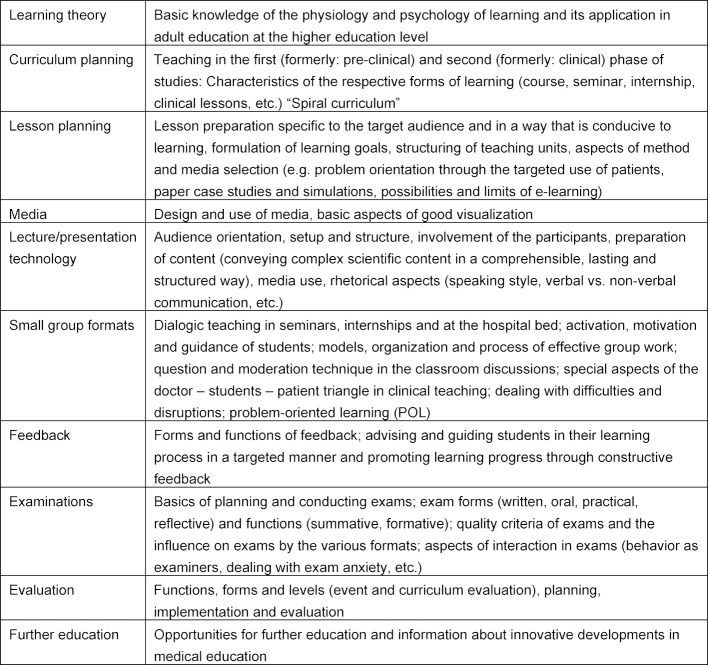
Contents and thematic focus of medical didactic qualification measures [8]

**Table 2:**
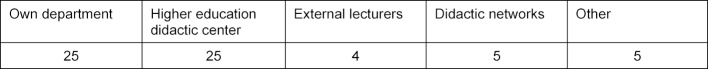
Providers of medical didactic workplace training (multiple answers possible)

**Table 3:**
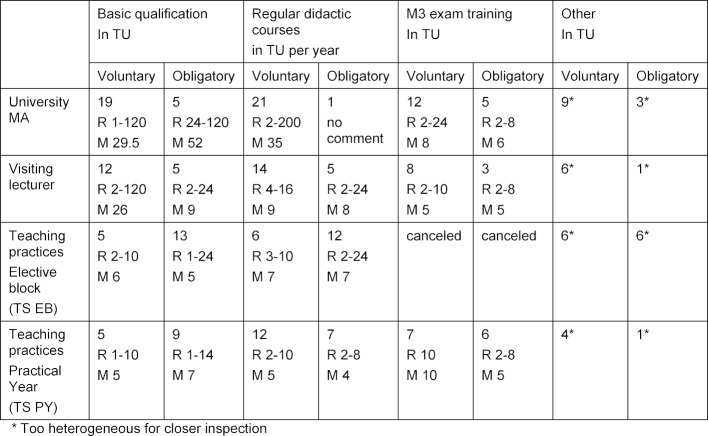
Number of General Practice higher education sites that have reported medical didactic offers including the range (R) of the teaching units offered (TU=45 min.) and the mean values (M)

**Table 4:**
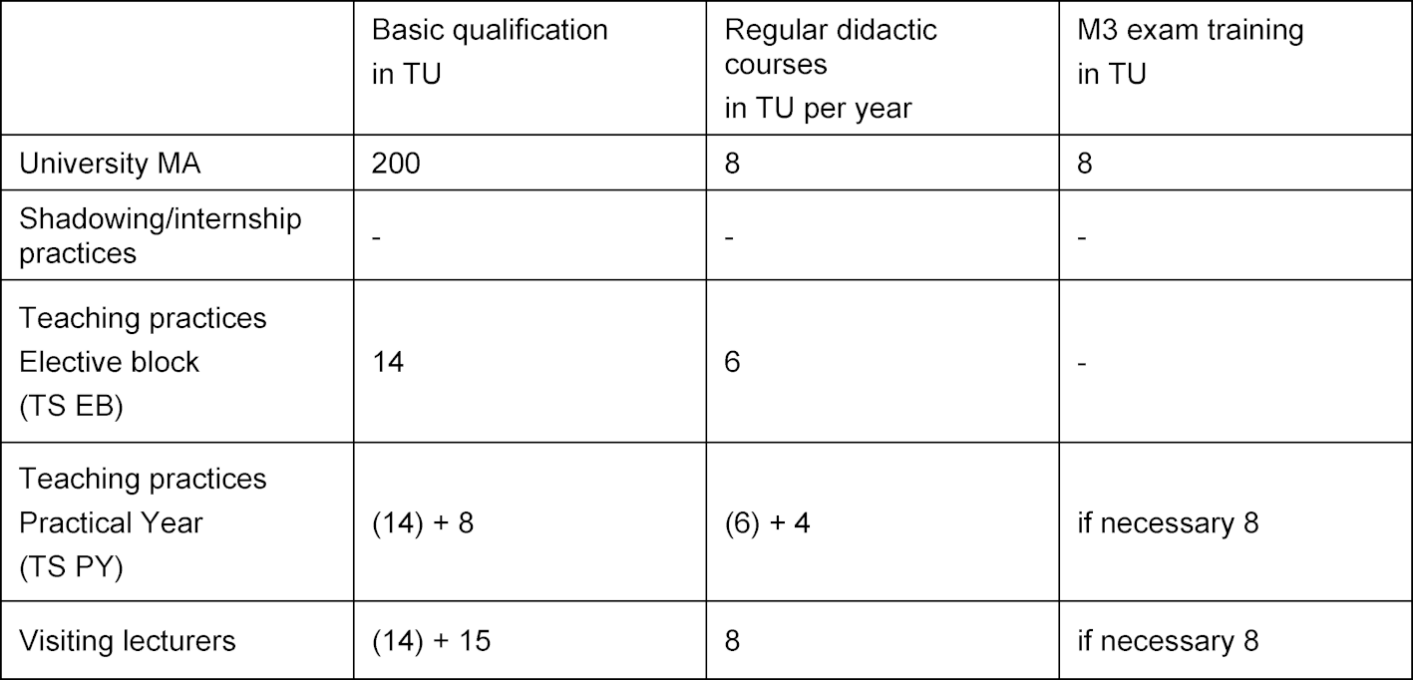
Overview of the recommended amount of time for didactic workplace training
